# Chemotherapy-induced peripheral neuropathy associated with docetaxel or nab-paclitaxel among patients with lung cancer: a prospective dual-center cohort study

**DOI:** 10.3389/fphar.2026.1795686

**Published:** 2026-06-11

**Authors:** Yalan Liu, Yurong Li, Xin Li, Hui Zhang, Xinfu Liu, Peng Chen

**Affiliations:** 1 Department of Thoracic Oncology, Tianjin Medical University Cancer Institute and Hospital, National Clinical Research Center for Cancer, Tianjin Key Laboratory of Cancer Prevention and Therapy, Tianjin’s Clinical Research Center for Cancer, Tianjin, China; 2 Department of Oncology, The Central Hospital of Shaoyang, Shaoyang, China

**Keywords:** CIPN, DOCETAXEL, Nab-Paclitaxel, pregabalin, questionnaire

## Abstract

**Objective:**

To compare the distinct clinical manifestations of chemotherapy-induced peripheral neuropathy (CIPN) in patients with different types of lung cancer treated with docetaxel or nab-paclitaxel, as well as to evaluate the post-intervention symptom improvement following therapeutic management.

**Methods:**

This prospective cohort study was conducted from September 2024 to August 2025 across two medical centers in China. Participants were lung cancer patients who had received treatment with either docetaxel or nab-paclitaxel. Chemotherapy-induced peripheral neuropathy (CIPN) was assessed using patient-reported outcomes from the European Organization for Research and Treatment of Cancer Quality of Life Questionnaire-CIPN 20-item scale (EORTC QLQ-CIPN20). Multivariable regression models were adjusted for patient baseline characteristics, tumor status, and medication-related features. Evaluations were performed using overlap propensity score weighting. Data analysis was carried out from September 2025 to December 2025.

**Results:**

Among the 850 participants, the mean (SD) age was 63.2 (10.5) years, with 385 patients (45.3%) receiving docetaxel and 465 patients (54.7%) receiving nab-paclitaxel. The docetaxel group primarily reported motor symptoms (e.g., leg weakness: 92 patients [34.2%]) and autonomic symptoms (e.g., blurred vision: 108 patients [40.1%]). In contrast, the nab-paclitaxel group predominantly reported sensory symptoms, such as numbness in the hands and feet (68 patients [25.3%]). The onset of patient-reported motor symptoms occurred earlier than sensory abnormalities, with median times of 2.3 weeks (95% CI, 1.3–5.4) in the docetaxel group and 0.8 weeks (95% CI, 0.6–1.2) in the nab-paclitaxel group. After adjustment using overlap propensity score weighting, patients in the docetaxel group had a lower risk of reported CIPN compared to those in the nab-paclitaxel group (HR, 0.65 [95% CI, 0.54–0.86]; *P* = 0.015). Patients treated with docetaxel reported fewer sensory discomforts compared to those receiving nab-paclitaxel (HR, 0.61 [95% CI, 0.49–0.89]; *P* = 0.001). However, the risk of reported motor symptoms (HR, 0.54 [95% CI, 0.44–0.69]; *P* = 0.061) and/or autonomic symptoms (HR, 0.87 [95% CI, 0.62–1.22]; *P* = 0.229) was not significantly lower in the docetaxel group than in the nab-paclitaxel group.

**Conclusion:**

In this cohort study of lung cancer patients, nab-paclitaxel was associated with more severe CIPN compared to docetaxel, regardless of patients’ clinical characteristics. Multivariate analysis identified taxane type, number of prior treatment cycles, treatment phase, and the use of pregabalin for intervention as independent factors associated with patient-reported CIPN. These findings may contribute to early intervention and therapeutic decision-making for CIPN during taxane-based treatment of lung cancer.

## Highlights

- This study reveals distinct chemotherapy-induced peripheral neuropathy (CIPN) profiles for docetaxel and nab-paclitaxel in lung cancer patients, with nab-paclitaxel associated predominantly with sensory symptoms and docetaxel linked more to motor and autonomic manifestations.
- The onset of patient-reported motor symptoms occurred earlier than sensory symptoms for both taxanes, highlighting the importance of comprehensive assessment using the EORTC QLQ-CIPN20 scale covering sensory, motor, and autonomic domains.
- After propensity score weighting, docetaxel was associated with a lower overall risk of patient-reported CIPN compared to nab-paclitaxel, and pregabalin intervention was found to alleviate sensory and motor symptoms.

## 1 Introduction

Based on the latest global cancer burden data released in 2025 by the International Agency for Research on Cancer (IARC), lung cancer currently ranks as the second most common malignancy globally and the leading cause of cancer-related mortality (Luo et al., 2025; Wu et al., 2024). Taxanes are among the most important chemotherapeutic agents for lung cancer. They are widely used in the adjuvant treatment of early-stage non-small cell lung cancer, neoadjuvant therapy for patients with locally advanced disease, and palliative care for those with advanced-stage disease (Wang et al., 2020). Furthermore, taxanes are also recommended for second-line treatment in patients with extensive-stage small cell lung cancer. Chemotherapy-induced peripheral neuropathy (CIPN) is one of the common adverse effects associated with taxanes, significantly impacting patients’ quality of life and even clinical outcomes. Approximately 60%–70% of patients receiving taxane-based therapy develop CIPN, which has become a major reason for premature treatment discontinuation (Luna-Rangel et al., 2025; Yu et al., 2025). Currently marketed taxanes with approved indications include solvent-based paclitaxel, liposomal paclitaxel, nab-paclitaxel, docetaxel, and polymeric micellar paclitaxel, among others (Xu et al., 2025). Among these, nab-paclitaxel and docetaxel are the two most widely used agents in clinical practice. Symptoms of CIPN typically emerge within the first 1–2 months of treatment, exhibit a cumulative dose effect, and may persist or even worsen after therapy cessation (Batash et al., 2025). As novel anti-tumor treatments continue to emerge and overall survival rates for lung cancer patients improve, CIPN induced by taxanes has become a treatment-related adverse event of significant concern for oncologists. Continuous exploration of new management strategies for this toxicity is underway in clinical practice to enhance patient treatment adherence. Therefore, a clear and comprehensive understanding of the clinical manifestations of taxane-associated CIPN is essential to avoid delayed recognition and missed optimal intervention timing.

Current grading systems for CIPN, such as the National Cancer Institute Common Toxicity Criteria for Adverse Events (NCI CTCAE) and the clinical version of the Total Neuropathy Score (Lee et al., 2025; Seth and Qureshi, 2023), primarily rely on physician assessment of patient-reported complaints combined with auxiliary clinical examinations, heavily dependent on the clinician’s subjective judgment. However, in practice, patient-reported symptoms are not always fully consistent with those documented in medical records by clinicians, a discrepancy influenced by factors such as the patient’s education level, communication skills, and the physician’s clinical experience. Given the logical necessity of incorporating patient-reported symptoms into CIPN assessment, a questionnaire-based approach was adopted from a practical standpoint to collect this data. Historically, taxane-related CIPN was predominantly considered a disorder affecting peripheral sensory nerves, primarily manifesting as tingling, numbness, and pain in the distal extremities (Nakajima et al., 2025). Traditional questionnaires often focused narrowly on assessing sensory system symptoms, overlooking other clinical manifestations of neuropathy involving motor and autonomic systems. In recent years, well-validated neuropathy assessment tools, such as the European Organization for Research and Treatment of Cancer (EORTC) Quality of Life Questionnaire Chemotherapy-Induced Peripheral Neuropathy 20-item scale (EORTC QLQ-CIPN20) (Liu et al., 2025; Boekhoudt et al., 2025), have been developed. These scales allow for a more detailed and multi-dimensional recording of the clinical manifestations of taxanes-induced peripheral neuropathy. A detailed phenotypic analysis of taxanes-related CIPN could facilitate personalized treatment selection and timely management of adverse events for lung cancer patients.

In previous clinical trials where efficacy was the primary endpoint, clinicians primarily assessed taxane-related neurotoxicity using the NCI CTCAE, finding a higher incidence of neurotoxicity associated with nab-paclitaxel compared to solvent-based paclitaxel, while no significant difference was observed between solvent-based paclitaxel and docetaxel (Burgess et al., 2021). However, these assessments largely focused on sensory system symptoms as the primary manifestation of CIPN, without incorporating evaluation of motor and autonomic neuropathy. Furthermore, these studies often involved different types of taxanes used at various treatment stages and employed inconsistent assessment scales, limiting comparability (Chen et al., 2022). The purpose of this study is to conduct a prospective cohort study across two different large tertiary hospitals, utilizing the EORTC QLQ-CIPN20 questionnaire to evaluate patient-reported CIPN and compare the characteristics associated with docetaxel and nab-paclitaxel.

## 2 Materials and methods

### 2.1 Study population and methods

This prospective cohort study recruited eligible lung cancer patients from two medical centers in China—namely, Tianjin Medical University Cancer Institute & Hospital and the Central Hospital of Shaoyang—between 1 September 2024, and 31 August 2025. Eligible patients were aged ≥18 years at diagnosis, had a pathological diagnosis of lung adenocarcinoma, squamous cell carcinoma, or small cell lung cancer, and were scheduled to receive or already receiving chemotherapy containing docetaxel or nab-paclitaxel at these centers, with an anticipated minimum of 4 treatment cycles. Exclusion criteria included a history of pre-existing peripheral neuropathy, treatment with other types of taxanes or no taxanes use, and failure to complete questionnaires following taxanes-based therapy. The attending physicians at each center determined the chemotherapy regimen based on standard clinical guidelines for lung cancer and individual patient circumstances. The study was approved by the Institutional Review Boards of both participating centers—Tianjin Medical University Cancer Institute & Hospital and the Central Hospital of Shaoyang (approval no. 2024-263). Written informed consent was obtained from all participants. Based on the anticipated incidence of patient-reported CIPN (approximately 60% in the docetaxel group and 75% in the nab-paclitaxel group based on preliminary data), with a two-sided alpha of 0.05 and power of 80%, we estimated that a minimum of 340 patients per group would be required. Considering a 15% dropout rate, we aimed to enroll at least 800 patients. The final cohort of 850 patients met this requirement. This study followed the Strengthening the Reporting of Observational Studies in Epidemiology (STROBE) guidelines (Skrivankova et al., 2021).

### 2.2 Data collection

The baseline enrollment time for patients was defined as the first day of the initial taxanes-based treatment cycle. At baseline and/or upon the patient’s return to the medical center for follow-up after each taxanes treatment cycle, we used the established assessment tool—the EORTC QLQ-CIPN20 questionnaire—to evaluate patient-reported CIPN. Patients completed the ratings by filling out paper questionnaires or using an electronic version (on mobile devices) in the interview room during their treatment period.

The validated EORTC QLQ-CIPN20 is a 20-item assessment tool that provides valuable information regarding CIPN-related symptoms and functional limitations experienced by patients. It includes sensory neuropathy symptoms (9 items, which also constitute the core component), motor neuropathy symptoms (8 items), and autonomic neuropathy symptoms (3 items) (Rattanakron et al., 2022). Each item is scored on a 4-point Likert scale, where 1 represents “not at all,” 2 represents “a little,” 3 represents “quite a bit,” and 4 represents “very much.” Standardized score = (raw score – item count)/(item count × 3) × 100. Higher scores indicate more severe neuropathy symptoms (Asogwa et al., 2025; Rahnea-Nita et al., 2024). We obtained the authorized Chinese version of this scale from the official website https://qol.eortc.org/. The survey also collected self-reported age and ethnic information from patients. Ethnicity was categorized as Han Chinese and other ethnic minorities (including Hui, Manchu, Mongolian, Russian, Uyghur, Miao, Tujia, and Zhuang), as disease characteristics and treatment responses may differ across ethnic groups. Since the two medical research centers are located in northern and southern China respectively, their patient populations are considered representative. To ensure the accuracy and completeness of the evidence, we reviewed the electronic health records of all enrolled patients in September 2025, covering clinical and treatment information.

The primary outcome of the study was the incidence of patient-reported CIPN as assessed by the EORTC QLQ-CIPN20 score. A patient-reported neurotoxic event was considered to have occurred when any single item score on the EORTC QLQ-CIPN20 scale exceeded 1 point. CIPN events occurring before the first questionnaire assessment were defined as left-censored, those occurring between two questionnaire assessments as interval-censored, and cases where no CIPN occurred by the last questionnaire assessment as right-censored. Corresponding statistical methods were applied to handle these censored data. Secondary outcomes included the sensory, motor, and autonomic subscale scores of the EORTC QLQ-CIPN20, as well as changes in these scores following pregabalin intervention.

The primary independent variable was the type of taxanes used (i.e., docetaxel or nab-paclitaxel). The dosage and duration of taxanes treatment were determined by the attending physician based on guideline recommendations and the patient’s specific condition. Demographic and clinical variables included: age, sex, smoking status, ethnicity (Han Chinese vs. ethnic minorities), body mass index (BMI), history of diabetes, tumor pathological type (squamous cell carcinoma, adenocarcinoma, small cell carcinoma), treatment phase (i.e., neoadjuvant, adjuvant, or palliative chemotherapy), chemotherapy regimen (monotherapy or combination therapy), number of chemotherapy cycles, and whether pregabalin intervention was administered.

### 2.3 Statistical analysis

Analysis of variance (ANOVA) and the chi-square (χ^2^) test were used to compare the baseline characteristics of patients receiving docetaxel or nab-paclitaxel. Linear mixed-effects models were employed to compare between-group differences in the EORTC QLQ-CIPN20 scores (total score, and sensory, motor, and autonomic subscale scores). The exposure factor, time, and their interaction were included in the models. As this was a secondary outcome, other covariates were not adjusted for in this model. The Kaplan-Meier curve using the nonparametric maximum likelihood estimation (NPMLE) method (McVittie et al., 2024) was applied to estimate the incidence of patient-reported CIPN. The generalized log-rank test was used for between-group comparisons of incidence rates, with a *P* value < 0.05 considered statistically significant.

To estimate the association between the two types taxanes and the incidence of patient-reported CIPN, we used a Cox proportional hazards regression model, as our data included left-censored, right-censored, and interval-censored observations. Three regression models were proposed: Model 1 included only the types of taxanes; Model 2 additionally adjusted for patient age, sex, ethnicity, region, body mass index (BMI), diabetes, pathological type, treatment phase, and chemotherapy regimen; Model 3 further extended Model 2 by including the number of cycles prior to CIPN reporting and the use of pregabalin. Using these three models, we estimated hazard ratios (HRs) and 95% confidence intervals (CIs) reflecting the independent association of each factor with patient-reported CIPN.

Furthermore, considering that the administration of taxanes in the enrolled population was non-random, we used propensity score methods (Davis et al., 2021) to reduce confounding effects. The generalized propensity score for the types of taxanes used was estimated via a multinomial logistic regression model incorporating patient age, sex, ethnicity, region, BMI, pathological type, and chemotherapy regimen. Data analysis was performed using generalized boosted modeling for overlap weighting (Wan et al., 2024), and covariate balance was assessed using the absolute standardized difference (ASD) for pairwise comparisons. Balance was considered good when the absolute ASD was close to 0.1. Subsequently, a Cox proportional hazards regression model with overlap propensity score weighting (Austin and Giardiello, 2025) was used to examine the relationship between types of taxanes and patient-reported CIPN on the EORTC QLQ-CIPN20, as well as other clinical outcomes. All analyses were conducted using IBM SPSS Statistics (version 27.0) and R software (version 4.2.3; R Project for Statistical Computing). Data analysis was performed from September 2025 to December 2025.

## 3 Results

### 3.1 Patient characteristics

**FIGURE 1:**
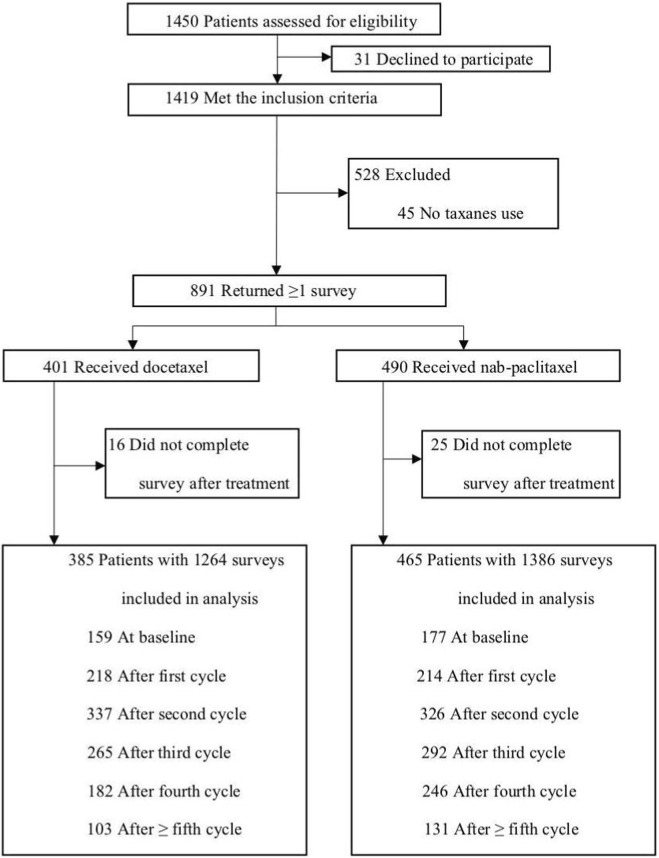
Participant selection flowchart. The reasons for 528 excluded patients are now categorized as follows: 312 patients did not meet inclusion criteria (including 187 with pre-existing peripheral neuropathy, 89 who received other types of taxanes, and 36 who were under 18 years of age); 145 patients declined to participate; and 71 patients were excluded due to incomplete questionnaire data.

A total of 850 lung cancer patients who received taxanes-containing chemotherapy were included in the analysis. A flowchart detailing the screening process is presented in Figure 1. The reasons for 528 excluded patients are now categorized as follows: 312 patients did not meet inclusion criteria (including 187 with pre-existing peripheral neuropathy, 89 who received other types of taxanes, and 36 who were under 18 years of age); 145 patients declined to participate; and 71 patients were excluded due to incomplete questionnaire data. As per the study design, eligible lung cancer patients from the two research centers voluntarily enrolled after screening against the inclusion and exclusion criteria. They originated from 9 ethnic groups across 6 provinces in mainland China. Table 1 summarizes the baseline demographic and clinical characteristics of the cohort prior to propensity score weighting. Among the 850 participants, the mean (SD) age was 63.2 (10.5) years, with 385 patients (45.3%) receiving docetaxel and 465 patients (54.7%) receiving nab-paclitaxel. Statistically significant differences were observed between the two patient groups regarding geographic region, BMI, history of diabetes, treatment phase, chemotherapy regimen, and the use of pregabalin (Table 1).

**TABLE 1:** Demographic and clinical characteristics of the cohort according to the type of taxanes.

Characteristic	Patients, No. (%)			P Value
	Docetaxel (n = 385)	Nab-paclitaxel (n = 465)	Overall (N = 850)	
Age, mean (SD), y	63.4 (8.4)	62.3 (9.7)	63.2 (10.5)	0.077
Gender	​	​	​	0.252
Male	232 (60.3)	298 (64.1)	530 (62.4)	​
Female	153 (39.7)	167 (35.9)	320 (37.6)	​
Ethnicity	​	​	​	0.115
Han	360 (93.5)	446 (95.9)	806 (94.8)	​
Other minority group^a^	25 (6.4)	19 (4.1)	44 (5.2)	​
Geographic region	​	​	​	0.027
North China	201 (52.2)	278 (59.8)	479 (56.4)	​
South China	184 (47.8)	187 (40.2)	371 (43.6)	​
BMI, mean (SD)	23.5 (3.1)	24.3 (3.6)	24.0 (3.2)	<0.001
Medical history of diabetes mellitus	​	​	​	<0.001
No	334 (87.4)	439 (94.4)	773 (90.9)	​
Yes	51 (13.2)	26 (5.6)	77 (9.1)	​
Tumor pathological types	​	​	​	0.843
Adenocarcinoma	165 (42.9)	201 (43.2)	366 (43.1)	​
Squamous cell carcinoma	134 (34.8)	154 (33.1)	288 (33.9)	​
Small cell lung cancer	86 (22.3)	110 (23.7)	196 (23.0)	​
Treatment stage	​	​	​	0.010
Neoadjuvant	125 (32.5)	169 (36.4)	294 (34.6)	​
Adjuvant	56 (14.5)	95 (20.4)	151 (17.8)	​
Palliative	204 (53.0)	201 (43.2)	405 (47.6)	​
Chemotherapy regimens	​	​	​	<0.001
Combinations^b^	203 (52.7)	305 (65.6)	508 (59.8)	​
Monotherapy	182 (42.3)	160 (34.4)	342 (40.2)	​
Pregabalin use	​	​	​	0.007
Yes^c^	198 (51.4)	196 (42.2)	394 (46.4)	​
No	187 (48.6)	269 (57.8)	456 (53.6)	​

Abbreviation: BMI, body mass index (calculated as weight in kilograms divided by height in meters squared).

^a^ Including 8 ethnic minority groups: Hui, Manchu, Mongolian, Russian, uygur, miao, tujia and Zhuang.

^b^ Taxanes in combination with other chemotherapeutic drugs

^c^ Patients who defined as having been treated with oral pregabalin continuously for 1 month or more according to the manufacturer’s instructions

Of the total 2,650 questionnaires returned, 2,017 (76.1%) contained at least one item score >1 and were used for symptom-profile description. The remaining 633 questionnaires reported no CIPN symptoms (all items scored 1) and were excluded from this descriptive analysis. Because our primary objective was to characterize the spectrum and relative frequency of specific CIPN symptoms (sensory, motor, autonomic) among patients who experienced CIPN, we excluded these 633 symptom-free questionnaires from the descriptive symptom-profile analyses (e.g., radar charts and the calculation of individual symptom proportions). Including them would have artificially lowered the observed percentages of each symptom and obscured the true pattern of neuropathy among affected patients. For the patient-level overall CIPN prevalence (i.e., the proportion of patients who ever reported any CIPN symptom during the study period), we used the full cohort of 850 patients, regardless of questionnaire status. At the patient level, CIPN was reported by 69.9% (269/385) of patients in the docetaxel group and by 81.3% (378/465) in the nab-paclitaxel group.

Importantly, for all time-to-event analyses (Kaplan-Meier curves and Cox regression), we included all 850 patients, and those who never reported CIPN by the final assessment were appropriately right-censored, consistent with the censoring principles (left-censored, interval-censored, right-censored) described in the statistical methods.

### 3.2 Patient-reported CIPN following taxanes use

**FIGURE 2:**
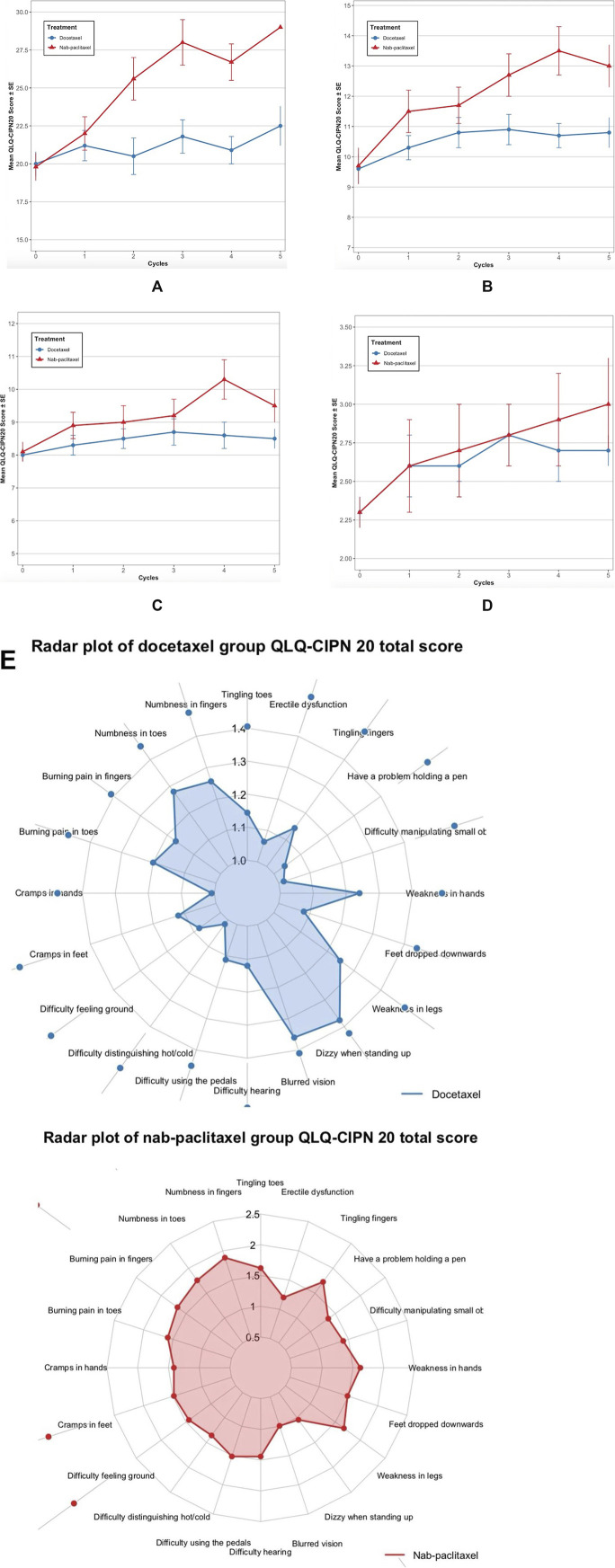
Association between types of taxanes and patient-reported neurotoxic effects **(A)** Absolute score of QLQ-CIPN 20 total score; **(B)** Absolute score of QLQ-CIPN sensory scale score; **(C)** Absolute score of QLQ-CIPN motor scale score; **(D)** Absolute score of QLQ-CIPN autonomic scale score. **(A–D)**, Dynamic changes in European Organization for Research and Treatment of Cancer Quality of Life Questionnaire: Chemotherapy-Induced Peripheral Neuropathy 20-item (QLQ-CIPN20) scores. Linear mixed-effects models showed that total QLQ-CIPN20 scores were significantly lower in the docetaxel group compared with the nab-paclitaxel group (6.8 [95% CI −9.2 to −4.4]; *P* < 0.001). Sensory scores (7.5 [95% CI −10.1 to −4.9]; *P* < 0.001) and later-phase motor scores (5.2 [95% CI −7.8 to −2.6]; *P* = 0.002) were also significantly lower in the docetaxel group. **(E)** Radar plot of docetaxel/nab-paclitaxel group QLQ-CIPN 20 total score.

Analysis of variance based on the linear mixed-effects model revealed that after taxanes treatment, the total EORTC QLQ-CIPN20 scores were consistently lower in the docetaxel group compared to the nab-paclitaxel group (Figure 2A). The sensory scores and the later-phase motor scores (Figures 2B,C) were significantly lower in the docetaxel group than in the nab-paclitaxel group, whereas the early-phase motor scores and autonomic scores were similar between the two groups (Figures 2C,D).

After four cycles of taxanes therapy, lung cancer patients receiving different taxanes reported distinct CIPN profiles. We summarized the scores for common adverse reactions and presented them in radar charts (Figure 2E). In the docetaxel group, the most frequently reported CIPN symptoms were primarily motor (e.g., lower limb weakness: 92 patients [34.2%]) and autonomic (e.g., blurred vision: 108 patients [40.1%]), accompanied by relatively mild sensory symptoms (numbness in hands and feet: 68 patients [25.3%]). In contrast, the nab-paclitaxel group predominantly exhibited numbness in the hands and feet related to sensory symptoms (268 patients, 82.5%), along with motor symptoms such as lower limb weakness (105 patients, 32.2%).

**FIGURE 3:**
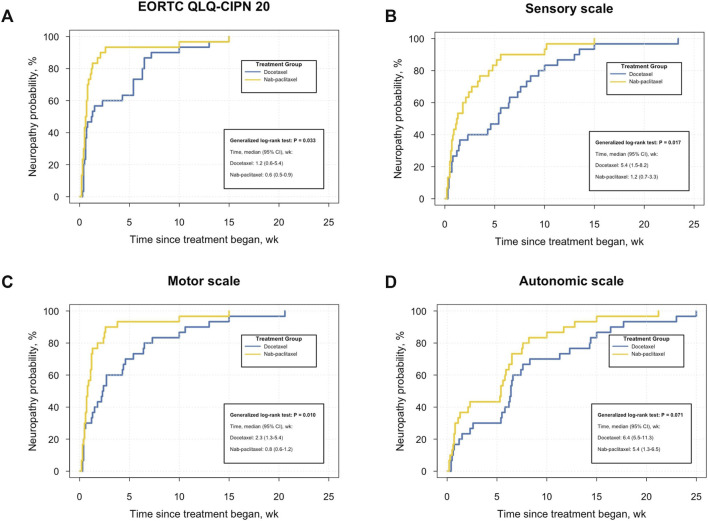
Incidence of patient-reported neuropathy estimated by the Kaplan-Meier curve using the nonparametric maximum likelihood estimation (NPMLE) method. **(A)** Overall CIPN incidence according to taxane type (docetaxel vs. nab‑paclitaxel); generalized log‑rank test P = 0.033. Median time to CIPN: 1.2 weeks (docetaxel) vs. 0.6 weeks (nab‑paclitaxel). **(B)** Sensory subscale: median time to sensory symptoms – 5.4 weeks (docetaxel) vs. 1.2 weeks (nab‑paclitaxel); P = 0.017. **(C)** Motor subscale: median time to motor symptoms – 2.3 weeks (docetaxel) vs. 0.8 weeks (nab‑paclitaxel); P = 0.010. **(D)** Autonomic subscale: median time to autonomic symptoms – 6.4 weeks (docetaxel) vs. 5.4 weeks (nab‑paclitaxel); P = 0.071.

Five weeks after treatment initiation (corresponding to the completion of cycle 2 in most patients), the incidence of patient-reported CIPN was significantly higher in the nab-paclitaxel group (92.3%) compared to the docetaxel group (63.5%; *P* = 0.033) (Figure 3A). Regarding the sensory subscale, the median time to patient-reported sensory symptoms was 1.2 weeks (95% CI, 0.7–3.3) after treatment start in the nab-paclitaxel group, which was significantly earlier than the 5.4 weeks (95% CI, 1.5–8.2) observed in the docetaxel group (*P* = 0.017), indicating a later onset in the latter group (Figure 3B). Notably, the onset of patient-reported motor symptoms occurred earlier than that of sensory symptoms. The median time to reporting motor symptoms was 2.3 weeks (95% CI, 1.3–5.4) in the docetaxel group and 0.8 weeks (95% CI, 0.6–1.2) in the nab-paclitaxel group, with a significant difference between the groups (*P* = 0.010) (Figure 3C). For autonomic symptoms, no significant difference was found between the two groups (*P* = 0.071). The median reporting times were 6.4 weeks (95% CI, 5.5–11.3) for the docetaxel group and 5.4 weeks (95% CI, 1.3–6.5) for the nab-paclitaxel group (Figure 3D).

The results of multivariate analysis indicated that the types of taxanes, the number of previous treatment cycles, the treatment phase, and the use of pregabalin for intervention were independent factors associated with patient-reported CIPN (Tables 2, 3). Compared to patients treated with nab-paclitaxel, those receiving docetaxel (HR = 0.62 [95% CI, 0.51–0.83]; *P* < 0.001) had a lower risk of developing CIPN. Similarly, on both the sensory and motor subscales, patients treated with docetaxel were less likely to report discomfort than those treated with nab-paclitaxel. The likelihood of reporting autonomic symptoms was comparable between the two groups (HR = 0.90 [95% CI, 0.72–1.12]; *P* = 0.359) (Table 2).

**TABLE 2:** Hazard risk difference in patient-reported CIPN induced by taxane types in patients with lung cancer (N = 850).

EORTC QLQ-CIPN20	Docetaxel vs. nab-paclitaxel		
	HR (95% CI)^a^ *P* Value	HR (95% CI)^b^ *P* Value	HR (95% CI)^c^ *P* Value
Total score	0.67 (0.53–0.76) <0.001	0.69 (0.56–0.85) <0.001	0.62 (0.51–0.83) <0.001
Sensory scale	0.45 (0.39–0.62) 0.024	0.57 (0.41–0.73) 0.025	0.68 (0.55–0.85) 0.016
Motor scale	0.73 (0.53–0.83) 0.013	0.75 (0.56–0.87) 0.002	0.54 (0.44–0.66) 0.031
Autonomic scale	0.87 (0.71–1.06) 0.156	0.81 (0.69–1.32) 0.383	0.90 (0.72–1.12) 0.359

Abbreviations: EORTCQLQ-CIPN20, European Organization for Research and Treatment of Cancer Quality of Life Questionnaire: Chemotherapy-Induced Peripheral Neuropathy 20-item; HR, hazard ratio.

^a^ Univariate model only include types of taxanes.

^b^Multivariable models accounted for baseline characteristics, including patient age, gender, ethnicity, geographic region, body mass index, medical history of diabetes mellitus, tumor pathological types, treatment stage and chemotherapy regimens.

^c^ Model further adjusted for treatment cycles and pregabalin use.

**TABLE 3:** Multivariate analysis using cox proportional hazards regression models.

Characteristics	Coefficient β	95% CI		*P* Value
		Lower limit	Upper limit	
Age	0.01	0.05	0.18	0.75
Gender	0.02	−0.14	0.17	0.51
Ethnicity (han compared to other minorities)	−0.12	−0.25	0.21	0.65
Geographic region	−0.34	−0.14	1.32	0.12
Body mass index (BMI)	0.04	−0.13	0.24	0.79
Tumour pathological types (compared to small cell lung cancer)				
Adenocarcinoma	−0.01	−0.17	0.25	0.96
Squamous cell carcinoma	−0.13	−0.24	0.19	0.54
Treatment cycles	−0.11	−0.27	−0.05	<0.01
Treatment stage (compared to adjuvant patients)				
Neoadjuvant	0.28	0.23	0.87	0.02
Palliative	0.17	0.05	0.39	<0.01
Chemotherapy regimens (compared to monotherapy)				
Combinations	0.08	−0.05	0.28	0.31
Types of taxanes (compared to nab-paclitaxel)				
Docetaxel	−0.52	−0.62	−0.23	<0.01
Pregabalin use	−0.63	−0.72	−0.38	<0.01

**FIGURE 4:**
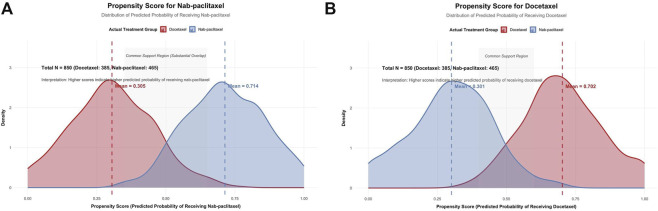
Propensity score distributions according the types of taxanes used. **(A)** Distribution of predicted probability of receiving docetaxel. The mean propensity score is 0.301 for the docetaxel group and 0.702 for the nab‑paclitaxel group. **(B)** Distribution of predicted probability of receiving nab‑paclitaxel. The mean propensity score is 0.305 for the docetaxel group and 0.714 for the nab‑paclitaxel group. The common support region shows substantial overlap.

**FIGURE 5:**
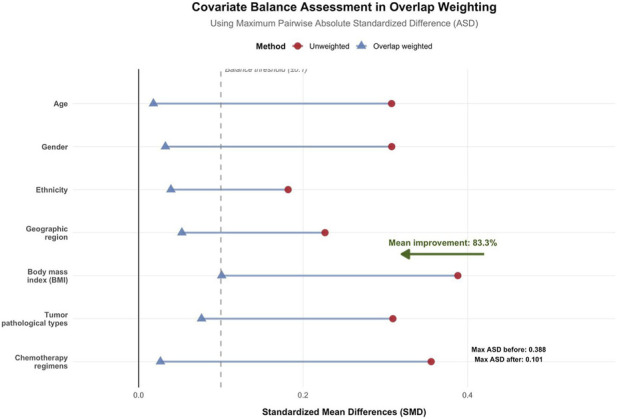
Covariate balance assessment in overlap weighting using maximum pairwise absolute standardized difference (ASD).

The propensity score distribution for different types of taxanes are shown in Figure 4. The weighted effective sample sizes were 383 for the docetaxel group and 468 for the nab-paclitaxel group (rounded to the nearest integer). After overlap propensity score weighting (Table 4; Figure 5), the risk of patient-reported CIPN was lower in the docetaxel group (HR, 0.65 [95% CI, 0.54–0.86]; *P* = 0.015) compared to the nab-paclitaxel group. Similarly, regarding sensory symptoms, patients receiving docetaxel (HR, 0.61 [95% CI, 0.49–0.89]; *P* = 0.001) reported less sensory discomfort than those receiving nab-paclitaxel. However, the risk of reporting motor symptoms was not significantly lower in the docetaxel group (HR, 0.54 [95% CI, 0.44–0.69]; *P* = 0.061) compared to the nab-paclitaxel group. The same applied to autonomic symptoms (HR, 0.87 [95% CI, 0.62–1.22]; *P* = 0.229) (Table 5).

**TABLE 4:** Cohort characteristics after overlap propensity score–weighting.

Characteristics	Patients, %		
	Docetaxel	Nab-paclitaxel	*P value* ^a^
Age, mean (SD)	63.1 (9.9)	63.5 (9.4)	0.55
Gender			0.90
Male	62.4	62.8	​
Female	37.6	37.2	​
Ethnicity			0.86
Han	93.8	94.1	​
Other minority group	6.2	5.9	​
Geographic region			0.83
North China	53.5	54.3	​
South China	46.5	5.7	​
Body mass index^b^	23.6 (5.4)	24.1 (3.6)	0.12
Tumour pathological types			0.98
Adenocarcinoma	43.1	43.5	​
Squamous cell carcinoma	34.4	33.7	​
Small cell lung cancer	22.5	22.8	​
Chemotherapy regimens			0.88
Monotherapy	53.6	54.1	​
Combinations	46.4	45.9	​

^a^ Values are weighted percentages based on overlap propensity score weighting. The weighted effective sample sizes were 383 for the docetaxel group and 468 for the nab-paclitaxel group (rounded to the nearest integer). Group comparisons were performed using weighted chi-square tests for categorical variables and weighted ANOVA for continuous variables

^b^ Calculated as weight in kilograms divided by height in meters squared

**TABLE 5:** Overlap-weighted association between taxane types and CIPN among patients with lung cancer (N = 850).

EORTC QLQ-CIPN20	Docetaxel vs. nab-paclitaxel		
	HR (95% CI)^a^ *P* Value	HR (95% CI)^b^ *P* Value	HR (95% CI)^c^ *P* Value
Total score	0.65 (0.51–0.79) 0.024	0.69 (0.52–0.81) <0.001	0.65 (0.54–0.86) 0.015
Sensory scale	0.41 (0.29–0.58) 0.016	0.56 (0.43–0.76) 0.027	0.61 (0.49–0.89) 0.001
Motor scale	0.71 (0.55–0.86) 0.023	0.72 (0.57–0.89) 0.021	0.54 (0.44–0.69) 0.061
Autonomic scale	0.86 (0.71–0.94) 0.462	0.82 (0.61–1.25) 0.286	0.87 (0.62–1.22) 0.229

Abbreviations.: EORTCQLQ-CIPN20: European Organization for Research and Treatment of Cancer Quality of Life Questionnaire: Chemotherapy-Induced Peripheral Neuropathy 20-item; HR, hazard ratio.

^a^ Univariate model only include types of taxanes.

^b^ Multivariable models accounted for baseline characteristics, including patient.

age, gender, ethnicity, geographic region, medical history of diabetes mellitus, tumor pathological types, treatment stage and chemotherapy regimens.

^c^ Model further adjusted for treatment cycles and pregabalin use.

## 4 Discussion

In this prospective, dual-center cohort study of lung cancer patients, we found that patients receiving different types of taxanes therapy reported distinct CIPN profiles. Patients in the nab-paclitaxel group most frequently reported symptoms related to sensory disturbances, such as numbness in the hands and feet, whereas those in the docetaxel group primarily reported motor symptoms (e.g., lower limb weakness) and autonomic symptoms (e.g., blurred vision) (Accordino et al., 2024). Furthermore, the risk of motor and autonomic symptoms was not low for either taxane, and the onset of motor symptoms even preceded that of sensory symptoms in some patients. After controlling for general patient characteristics and tumor-related factors (such as pathological type, treatment phase, and number of treatment cycles), lung cancer patients treated with docetaxel had a lower risk of reporting CIPN compared to those treated with nab-paclitaxel. Following intervention with pregabalin, the occurrence of sensory and motor neuropathy symptoms was reduced in both taxane groups, while the effect on autonomic symptoms was not pronounced. Although pregabalin was associated with symptom improvement in our study, its routine use as a premedication is not currently supported by clinical guidelines and may be limited by its side effect profile. Future randomized trials are needed to evaluate its prophylactic potential.

Our study is the first to suggest that lung cancer patients treated with two commonly used chemotherapeutic agents—docetaxel and nab-paclitaxel—exhibit distinct neurotoxicity profiles. The most common manifestation of taxane-associated CIPN is numbness in the hands and feet related to sensory symptoms. Some studies have indicated that motor and autonomic adverse events may even occur before sensory symptoms emerge following taxanes treatment. Indeed, the symptoms primarily reported by patients in the docetaxel group were motor (lower limb weakness) and autonomic (blurred vision). All patients in the docetaxel group received dexamethasone premedication according to standard practice, whereas those in the nab-paclitaxel group did not. This difference may contribute to the higher frequency of motor and autonomic symptoms reported in the docetaxel group. We speculate that this may be related to the premedication with dexamethasone before chemotherapy, as corticosteroid-induced polyneuropathy/myopathy is a muscular disorder associated with glucocorticoid use or elevated endogenous hormone levels (Kim et al., 2022). Long-term use of corticosteroids may lead to decreased muscle strength in cancer patients, affecting their mobility and tolerance, which could subsequently influence further cancer treatments such as chemotherapy and radiotherapy (Goodman et al., 2023). Previous clinical studies on taxane-related neurotoxicity have predominantly focused on sensory symptoms, with insufficient attention paid to the manifestations of motor and autonomic symptoms (Trinh et al., 2025). Once CIPN occurs, there are no specific therapeutic agents or approaches, often resulting in treatment delays or discontinuation, which further impacts patient outcomes. Therefore, this study also aims to draw more clinical attention to the delayed neurotoxicity induced by taxanes, enabling earlier detection and intervention.

With the continuous emergence of targeted therapies, immunotherapies, and antibody-drug conjugates in recent years, the survival of advanced lung cancer patients has been prolonged, making quality of life an increasingly important consideration (Coleman et al., 2023). Previous studies assessing CIPN often relied on the subjective judgment of investigators, introducing a degree of reporting bias. In recent years, patient-reported outcomes have gained prominence in numerous clinical trials and have even been incorporated as primary endpoints (Li et al., 2023). Studies such as that by Kaitlin Chen et al. (Chen et al., 2026) have shown that the minimal clinically important differences in CIPN assessments vary depending on the scales and evaluation tools used in specific populations. Since clinicians’ subjective assessments often underestimate patients’ CIPN symptoms, there is a discrepancy between patient-reported outcomes related to CIPN and medical records. Currently, the most widely used questionnaire for assessing patient-reported CIPN is the EORTC QLQ-CIPN20, which is also one of the most globally utilized surveys (Prutianu et al., 2022). In this study, we applied a version of the questionnaire adapted for the Chinese population, obtained from the official website, to better reflect the most authentic feedback on adverse events during the use of the two drugs. This approach aims to assist clinicians and patients in identifying taxane-associated CIPN in clinical practice.

## 5 Limitations

Despite employing methodologically rigorous statistical models, such as the Cox proportional hazards model with overlap propensity score weighting and multivariable regression models, this study has several limitations. First, as an observational cohort study rather than a randomized clinical trial, our findings may be subject to unmeasured confounding, despite the use of robust propensity score-based methods. The autonomic subscale of the EORTC QLQ-CIPN20 consists of only three items and is known to have a lower internal consistency; missing data were also observed for certain items, which may affect the reliability of this subscale. Factors such as the expertise of the researchers, the source of participants, and the consistency in questionnaire administration may introduce unpredictable confounding variables. Second, a notable number of patients did not complete the questionnaires as planned at each treatment cycle, resulting in missing data. This attrition bias may have affected the reliability of the observed outcomes to some extent. Third, the study did not uniformly adjust for participants’ educational and cultural backgrounds, which could influence the accuracy of patient-reported responses when completing the questionnaires. Future studies should consider stratifying by education level to further explore this potential bias.

## 6 Conclusion

The findings of this cohort study suggest that lung cancer patients treated with docetaxel and nab-paclitaxel may present with distinct CIPN profiles. Nab-paclitaxel is associated with higher CIPN scores and a greater incidence of CIPN compared to docetaxel. Beyond sensory neuropathy symptoms, the risk of patient-reported motor and autonomic symptoms is not low with either of these two types of taxanes. Intervention with pregabalin may help reduce the occurrence of sensory and motor neuropathy symptoms to some extent. These findings could contribute to the early detection and management of taxane-associated CIPN in lung cancer patients.

## Data Availability

The datasets presented in this study can be found in online repositories. The names of the repository/repositories and accession number(s) can be found in the article/supplementary material.
